# Intestinal Microbiota and Celiac Disease: Cause, Consequence or Co-Evolution?

**DOI:** 10.3390/nu7085314

**Published:** 2015-08-17

**Authors:** María Carmen Cenit, Marta Olivares, Pilar Codoñer-Franch, Yolanda Sanz

**Affiliations:** 1 Microbial Ecology, Nutrition & Health Research Group, Institute of Agrochemistry and Food Technology, National Research Council (IATA-CSIC), Avda. Agustín Escardino, 7, 46980 Paterna, Valencia, Spain; E-Mail: m.olivares@iata.csic.es; 2 Department of Pediatrics, Dr. Peset University Hospital, Avda. Gaspar Aguilar, 80, 46017 Valencia, Spain; E-Mail: pilar.codoner@uv.es; 3 Department of Pediatrics, Obstetrics and Gynecology, University of Valencia, Av Blasco Ibáñez, 13, 46010 Valencia, Spain

**Keywords:** microbiota, celiac disease, gluten-free diet, dysbiosis

## Abstract

It is widely recognized that the intestinal microbiota plays a role in the initiation and perpetuation of intestinal inflammation in numerous chronic conditions. Most studies report intestinal dysbiosis in celiac disease (CD) patients, untreated and treated with a gluten-free diet (GFD), compared to healthy controls. CD patients with gastrointestinal symptoms are also known to have a different microbiota compared to patients with dermatitis herpetiformis and controls, suggesting that the microbiota is involved in disease manifestation. Furthermore, a dysbiotic microbiota seems to be associated with persistent gastrointestinal symptoms in treated CD patients, suggesting its pathogenic implication in these particular cases. GFD *per se* influences gut microbiota composition, and thus constitutes an inevitable confounding factor in studies conducted in CD patients. To improve our understanding of whether intestinal dysbiosis is the cause or consequence of disease, prospective studies in healthy infants at family risk of CD are underway. These studies have revealed that the CD host genotype selects for the early colonizers of the infant’s gut, which together with environmental factors (e.g., breast-feeding, antibiotics, *etc.*) could influence the development of oral tolerance to gluten. Indeed, some CD genes and/or their altered expression play a role in bacterial colonization and sensing. In turn, intestinal dysbiosis could promote an abnormal response to gluten or other environmental CD-promoting factors (e.g., infections) in predisposed individuals. Here, we review the current knowledge of host-microbe interactions and how host genetics/epigenetics and environmental factors shape gut microbiota and may influence disease risk. We also summarize the current knowledge about the potential mechanisms of action of the intestinal microbiota and specific components that affect CD pathogenesis.

## 1. Introduction

Celiac disease (CD) is a chronic immune-mediated inflammatory disease affecting the small bowel, triggered by gluten ingestion in genetically susceptible individuals. Even though CD is an infra-diagnosed disorder, it is currently considered the most common food intolerance, affecting approximately 1% of European ancestry individuals.

CD is a complex multifactorial disorder involving both genetic and environmental factors. For a long time, the only securely established genetic factors contributing to CD risk were various genetic variants located within the HLA region (those encoding the HLA-DQ2/DQ8 heterodimers) [1]. Gluten peptides presented by HLA-DQ2/DQ8 heterodimers stimulate HLA-DQ2 and HLA-DQ8 restricted T cells, triggering a complex immune response involving both the innate and adaptive immune system. With the introduction of GWAS (*genome wide association studies*) and the Immunochip study, an additional 39 non-HLA regions of susceptibility have been associated with CD development, some of which are shared with other autoimmune diseases [2,3,4,5,6,7]. CD is a complex immune-related disorder with the best characterized genetic component; however, only an approximate 31% of its heritability has been explained so far, suggesting that other genetic factors besides gene–gene and gene–environment interactions might be involved in disease development [1]. Interestingly, most of those chromosome regions associated with CD predisposition contain genes with immune related functions and some CD susceptibility genes and/or their altered expression play a role in bacterial colonization and sensing. Studies have also revealed an altered expression of non-specific CD risk-genes involved in host–microbiota interactions in the intestinal mucosa of CD patients, such as those of Toll-like receptors (TLRs) and their regulators [8]. Furthermore, 81% of CD associated genetic variants are located in noncoding regions of the genome [9], suggesting that one of the main mechanisms by which genetic variation could have an impact on CD is by affecting the gene expression levels. Thus, the altered expression of CD-risk genes, as well as other non-specific CD genes triggered by genetic and epigenetic factors, may contribute to disturbing the host–microbiota interaction, and shift immune balance in CD subjects. Similar findings have been reported for inflammatory bowel disease (IBD) [10], a disorder characterized by a deregulated immune response against the microbiota, triggered by specific genetic determinants [11].

CD commonly appears in early childhood after the first exposures to dietary gluten, which is its main environmental trigger. However, there are increasing numbers of subjects experiencing CD onset in early and late adulthood [12], which suggests that additional environmental factors must play a role in CD development. In fact, other environmental factors that influence the early gut microbiota composition such as birth delivery mode and milk-feeding type, intestinal infections and antibiotic intake, have also been associated with the risk of developing CD [13,14,15,16,17,18]. Thus, a number of epidemiological studies indicate that several perinatal factors participate in conjunction to modulate CD risk.

Many complex immune-mediated diseases have been linked to changes in the composition of the gut microbiota and its genome (microbiome), including CD [19,20,21,22,23]. It has also recently been observed that the microbiota differs among the different subgroups of CD patients stratified according to specific clinical manifestations [24]. Moreover, although the vast majority of patients diagnosed with CD respond to a GFD there is a subgroup of CD patients that do not show clinical improvement after adherence to a GFD [24]. In particular, patients suffering persistent symptoms on a long-term GFD also show an altered microbiota composition [25]. CD14 is, together with TLR-4, involved in the recognition and signal transduction of bacterial endotoxin or lipopolysaccharide, a major component of the bacterial cell wall of Gram-negative bacteria. The CD14/TLR-4 complex, upon binding, triggers innate host defense mechanisms, such as the release of pro-inflammatory cytokines. Soluble CD14 (sCD14) is commonly used as an indicator of innate immunity cell activation in response to mucosal translocation of Gram-negative bacteria or their components [26]. Interestingly, it has recently been reported that sCD14 protein seropositivity is increased in untreated CD patients [27]. These increased sCD14 serum levels in CD could be the consequence of translocation of commensal intestinal bacteria, which could aggravate CD pathogenesis. Taken together, all this evidence suggests a role for the microbiota in disease manifestation, pathogenesis and risk. It also opens up the possibility of finding new strategies for alleviating the symptoms of specific patient subgroups or reducing the risk of the disease by intentional modulation of the intestinal microbiota.

Here, we review the current knowledge about host–microbe interactions and how host genetics/epigenetics and environmental factors shape the gut microbiota and may influence disease risk. We also summarize the current understanding of the potential mechanisms of action of the intestinal microbiota and its specific components in CD pathogenesis.

## 2. Host Immune–Microbiota Interactions

Initially, microbes were viewed solely as pathogens that cause and propagate infectious diseases. Nowadays, it is well established that human beings harbor microbial communities with key beneficial health functions. Indeed, most of these microbes are commensal and play an important role in our metabolism, mediating food digestion, and in the development and polarization of immune responses, preventing pathogens from invading our body [25]. The microbiota, namely the microbial communities harbored by the host, outnumber human cells by a factor of 10 and encode hundreds of genes that are absent in the human genome [28].

The human immune system and gut microbiota clearly interact with each other in such a way that one shapes the other to a large extent. The immune system plays a crucial role in protecting humans from invading pathogens and in maintaining the self-tolerance. However, in the case of autoimmunity, the breakdown of physiological mechanisms responsible for maintaining tolerance to self-antigens leads the immune system to attack the body’s own tissues. It has been suggested that dysbiosis may affect autoimmunity by altering the balance between tolerogenic and inflammatory members of the microbiota and, therefore, the host immune response.

The human immune system has developed different mechanisms to tolerate commensal microbes and prevent pathogens invading the host [29]. In this respect, the microbiota increases the epithelial barrier function through the production of different metabolites, such as short-chain fatty acids (SCFAs) and mucus. The microbiota also promotes the production of antimicrobial molecules such as regenerating islet-derived protein III (REGIII)-γ and REGIII-β by epithelial cells in the intestine [29]. Researchers report that germ-free mice and mice treated with broad-spectrum antimicrobials showed a reduced proliferation of intestinal epithelial cells (IECs) and also a lower production of antimicrobial peptides [30,31]. Furthermore, this host–microbiota relationship also ensures the establishment of immune homeostasis so that the host’s immune system does not attack the commensal microbes. Pattern-recognition receptors (PRRs), including TLRs, located on IECs and also on antigen presenting cells (APCs) at the interface between the host and microbiota, recognize and integrate signals from microbial associated motifs and regulate intestinal barrier function and immune responses [23]. The inflammatory response triggered by TLR signaling can be further controlled either by intracellular regulators, which can inhibit TLR signaling pathways, or by the production of anti-inflammatory cytokines that are also modulated by the microbiota [29]. In addition, several studies have found that different functions of macrophages, dendritic cells and neutrophils, which are an essential part of the innate immune system, are modulated by the microbiota [32,33]. Furthermore, the gut microbiota seem to play a critical role in differentiating a second type of Natural Killer (NK) cells (IL-22^+^NKp46^+^) which belongs to the group of innate lymphoid cells (ILCs) with an important role in regulating homeostasis and inflammation [34].

**Figure 1:**
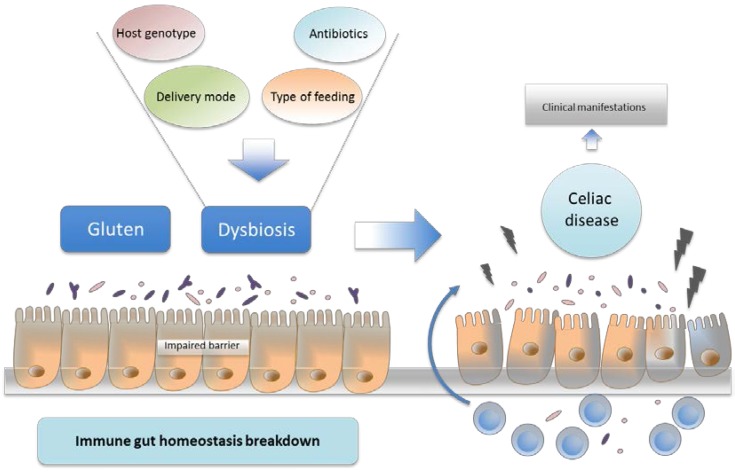
Proposed model for celiac disease (CD) pathogenesis. Specific host genetic makeup and environmental factors could promote the colonization of pathobionts and reduce symbionts, thus leading to dysbiosis. Dysbiosis may contribute to disrupting the immune homeostasis and gut integrity, thereby favoring CD onset and aggravating the pathogenesis.

Other studies also support a role of the gut microbiota in the development and function of the adaptive immune system. Specific microbial groups are associated with the initiation of specific T cell responses; for instance, *Bacteroides fragilis* induces the differentiation of Treg cells, promoting an anti-inflammatory immune response [35]. Furthermore, *Clostridium* spp., belonging to clusters IV and XIVa, have also been associated with the differentiation of CD4^+^ T cells into IL-10 producing-Treg cells in the germ-free mice intestinal mucosa, colonized with a specific bacterial mixture of clostridia [36]. Segmented filamentous bacteria (SFB) comprise a group of Gram-positive clostridia-related bacteria that strongly stimulate immune responses. Indeed, SFB have been associated with a pro-inflammatory response, inducing the differentiation of naïve CD4^+^ T cells into Th17 cells [37]. SFB mediate a state of controlled inflammation, which primes the gastrointestinal tract to be ready for pathogen invasion, thus protecting the host against acute infections (e.g., *Citrobacter rodentium*, a bacterial pathogen affecting animals that causes acute intestinal inflammation similar to enteropathogenic *Escherichia coli* (EPEC) in humans) [37]. However, SFB colonization could also lead to adverse host effects. SFB can therefore be considered as examples of pathobionts, which are potentially pathogenic microorganism comprising the indigenous microbiota but that may contribute to disease under certain circumstances (triggered by environmental or genetic factors), possibly involving increased numbers or adaptive mutations [38,39,40,41]. Therefore, the specific host genetic makeup and environmental factors could contribute to promoting or preventing the colonization of particular microorganisms, influencing their numbers and virulence features, thereby shaping a pro-inflammatory or anti-inflammatory intestinal milieu. CD is well characterized by an upregulated Th1 immune response (increased IFN-γ) and consequently a Th1 polarized inflammation even observed in patients following a GFD. Recent studies have suggested that the increased expression of Th1 cytokines observed in CD may have partly resulted from the microbiota imbalance and/or the altered expression of PPRs which could play a role in shifting responsiveness towards Th1-type immunity [8,42,43]. Human genetics and host-associated microbial communities have been related independently to a wide range of chronic diseases, including CD. We now know that environmental factors and host genetics interact to regulate microbiota acquisition and to maintain healthy gut microbiota stability [44,45]. In turn, these three components seem to interact strongly, maintaining gut integrity and immune gut homeostasis. The disruption of gut integrity and disturbance of immune gut homeostasis caused by modifying one or more of the three interacting components may trigger the development of diseases such as CD (Figure 1) [46].

## 3. Host Genetics and Intestinal Microbiota

Although gut microbiota composition shows large inter-individual variability, family members have more similar microbiota than unrelated individuals and, indeed, the same bacterial strains are shared among family members [47,48,49]. These similarities between the microbiota of related members most likely reflect the influence of the host genetic makeup although the shared environmental factors have also been shown to have an effect. Over 30 years ago, a study reported that the fecal microbiota of monozygotic human twins was much more similar than that of dizygotic twins [47].

Some years ago, researchers tested whether specific taxa co-segregated as quantitative traits linked to genetic markers using quantitative trait loci (QTL) analysis in mice [50]. The QTL detection approach revealed 18 host-associated QTLs having a linkage with the abundance of 26 specific microbial taxa. In addition, they established that one QTL is often associated with more than one taxon, indicating that human genetics may strongly influence the microbiota community structure. Interestingly, a QTL associated with abundance of specific bacterial taxa (the genus *Lactococcus* and the family Coriobacteriaceae) was found to contain important genes for mucosal immunity: Irak3 (encoding IL-1 receptor-associated kinase 3, which modulates the Myeloid differentiation primary response gene 88 (*Myd*88)-dependent TLR-2 pathway), *Lyz*1 and *Lyz*2 (two primary mouse lysozyme genes), *Ifng* (the interferon-γ gene) and *Il22* (the interleukin-22 gene) [50].

In addition, candidate gene approaches showed that a single host gene can have a remarkable effect on the microbiota composition [51]. Not surprisingly, most of the genes that have been identified as genes associated with microbiota changes encode factors involved in bacterial sensing and immune reactions, while some others are involved in metabolism [52]. The first human gene for which variation was shown to influence the gut microbiota was Mediterranean fever (*MEFV*) [51]. Specifically, this study revealed that changes in the human gut microbiota are associated with a single mutation in *MEFV*, which leads to a hereditary autoinflammatory disorder affecting people with Mediterranean ancestors, the so-called familial Mediterranean fever.

PRRs as well as antimicrobial peptides are key factors controlling the intestinal microbiota composition. Indeed, deficiencies in these genes lead to changes in the composition of the gut microbiota [52]. Animal studies have indicated that genes coding for inflammasome-related proteins, which are also involved in the recognition of microbial or other damage signals, influence intestinal microbiota composition and colitis development. Actually, deficient mice in the pyrin 6 member of the nucleotide-binding oligomerization domain-like receptor (N*lrp*6) showed different fecal microbiota characterized by increased representation of Bacteroidetes (Prevotellaceae) and TM7, reduced IL-18 production by epithelial cells and exacerbation of colitis induced by exposure to dextran sodium sulfate [53]. Very recently, a study has reported that NLRP6 inflammasome regulates goblet cell mucus secretion, showing that NLRP6 inflammasome-deficient mice are highly susceptible to persistent infection since they are unable to clear enteric pathogens from the mucosal surface [54]. Some years ago, the capacity of interferon (IFN) signaling pathways to modulate the microbiota composition was demonstrated in mice [55]. Thompson *et al.* revealed that the microbiota was less stable in IFN regulatory factor 9 (*I*rf9) knock out mice, which is primarily involved in type I IFN signaling than in control mice [55].

GWAS have revealed that genes involved in innate and adaptative immunity are associated with inflammatory diseases [5,56,57]. Interestingly, several of these genes have been shown to have a role in shaping the microbiota. Particularly, many of the IBD-susceptibility genes regulate host–microbial interactions [57]. Some of these loci are involved in bacterial sensing and immune reactions and might contribute to explaining the relationship between IBD and intestinal dysbiosis. For instance, nucleotide-binding oligomerization domain containing 2 (NOD2) is an intracellular sensor of bacterial peptidoglycan strongly expressed in Paneth cells, regulating their function, which is to release granules containing antimicrobial peptides in response to bacteria [58]. *NOD2* has been identified as a susceptibility gene for Crohn’s disease and different NOD2 polymorphisms have been associated with loss-of-function of the protein. Recently, a study focusing on IBD revealed a significant association between NOD2 risk allele counts and increased relative abundance of Enterobacteriaceae [59]. Furthermore, NOD2-deficient mice display a diminished ability to kill bacteria and increased loads of commensal bacteria, demonstrating that NOD2 is essential for regulating intestinal microbiota [60]. Subsequent studies have demonstrated that NOD2 genotypes also affect human microbial composition [61]. NOD2-deficient mice displayed increased responses to TLR stimulation, which might mirror the situation in genetically susceptible individuals [62]. Therefore, it is tempting to speculate that NOD2 polymorphisms could increase susceptibility to Crohn’s disease by suppressing TLR homeostasis, which would trigger a pathogenic response to the commensal microbiota. A recent study focusing on IBD demonstrated reproducible effects of a number of host genes on the microbiome taxonomic structure across two or more cohorts; some of the studied genes have known involvement in microbial handling while others are of unknown function [59]. Interestingly, beside NOD2, tumor necrosis factor (ligand) superfamily member 15 (TNFSF15) and subunit beta of interleukin 12 (IL12B) showed significantly conserved directionality effects on bacterial taxa between at least one pair of studies. A functional enrichment analysis showed that genes regulating the innate immune response, the JAK-STAT pathway and other immunity-related pathways, seem to be related with microbiome features [59]. Most likely some of those genotype-microbiome associations may be IBD-independent and relevant to individuals with other diseases such as CD.

Another study described that the β-1, 4-n-acetyl-galactosaminyltransferase 2 (*B4galnt2*) gene, encoding a mucosal surface glycan with an important role in host–microbiota interaction, influences the abundance of specific bacterial taxa microbiota composition [63]. A recent study reported the link between Cystic fibrosis transmembrane conductance regulator (*CFTR*) gene variants and shifts in fecal microbiota [64]. Furthermore, a rare polymorphism located within the immunity-related GTPase family M (*IRGM*) gene (involved in autophagy and with a potential role in microbiota homeostasis) is reported to show a significant correlation with a *Prevotella*-predominant enterotype [65].

Another recent study has compared the microbiota of 416 twin pairs, identifying many specific members of the gut microbiota whose abundances were influenced by the host genetic makeup, while other members seem to be determined by environmental factors [44]. Specifically, the family Christensenellaceae showed the highest heritability, forming a co-occurrence network with other heritable bacteria and Archaea in lean individuals; however, Bacteroidetes seem to be mostly determined by the environment. Interestingly, the study showed that Christensenellaceae was enriched in lean individuals, and was associated with reduced weight gain in mice. Therefore, the results indicate that host genetics influence gut microbiome composition, and may do so in ways that impact host metabolism [44]. All the above evidence would indicate that host genetic factors influence both the composition of gut microbiota and disease risk.

To date, several loci have been associated with microbiota composition; however, it is worth mentioning that it is as yet unknown how the complete human genome influences the microbiome. A variety of evidence suggests that a substantial number of genetic factors in humans may contribute with a relatively weak effect on the microbiota composition. Future studies should focus on analyzing all the host alleles underlying heritability of the gut microbiome as this would shed more light on the relationship between host genotype and microbiome composition.

## 4. CD Genetics and Intestinal Microbiota

CD is a disorder with a complex non-Mendelian pattern of inheritance, involving major histocompatibility complex (MHC) and non-MHC genes. The main genetic risk factor for CD falls within the MHC region, a region located on 6p21 responsible for the strongest association signals observed in most immune-mediated diseases, which contains hundreds of genes with immunological functions. Specifically, the alleles encoding human leukocyte antigen (HLA)-DQ2 have been identified as playing a key role in the genetic risk conferred by the MHC region. In fact, these HLA-associated alleles are much more frequently found in patients with CD (up to 95%) than in the general population (up to 35%). The main function of the MHC II molecules is to present bacterial antigens to T cells and to activate the immune system.

Some years ago, a prospective study in a cohort of 164 infants with a family history of the disease reported association between CD genetic risk (HLA-DQ genotype) and intestinal microbiota composition. In this study, the HLA-DQ2/8 genotype and the type of feeding (maternal or formula) were shown to influence, in conjunction, the intestinal microbiota composition [66]. In addition, specific decreases in *Bifidobacterium* spp. and *B. longum* and increases *Staphylococcus* spp. were associated with higher genetic risk of developing CD, regardless of milk-feeding type [66]. A recent microbiome analysis performed using next generation sequencing on a sub-cohort of 22 infants, all breast-fed and vaginally delivered, confirmed that the HLA-DQ genotype, in itself, influences the intestinal microbiota composition [67]. The high risk (HLA-DQ2 genotype) infant group showed an increased proportion of Firmicutes and Protebacteria and a reduction in Actinobacteria (including the genus *Bifidobacterium*) [67]. Furthermore, several studies based on different animal models have also indicated the presence of certain MHC polymorphisms that influence fecal microbiota composition [68,69].

To date, we have limited knowledge of the mechanisms by which the HLA-DQ genotype could selectively influence colonization and composition of the gut microbiota. The main function of MHC II molecules is to activate MHC restricted T cells. Therefore, we can speculate that different degrees of T cell activation, depending on the antigen presented to the T cells, could contribute to regulating the gut microbiota by enhancing B cell responses. These responses could involve the release of protective antibodies or promote T cell maturation into different effector cells such as Th1, Th2, Th17 or Foxp3^+^ Treg cells, the latter with immunosuppressive activity, which could contribute to developing tolerance towards the intestinal microbiota [70]. One murine study has supported this hypothesis, indicating that the repertoire of thymus-derived Treg cells is profoundly influenced by microbiota composition [71]. In turn, gut colonization dictated by the genotype could influence the risk of developing CD. Thus, De Palma *et al.* described an increased abundance of *Staphylococcus* spp. in the group of infants with higher genetic risk (HLA-DQ2) of developing CD. Staphylococcal superantigens bind directly to HLA class II molecules and strongly activate T cells. *HLA class II* polymorphisms can determine the strength of the superantigen HLA class II binding, by governing the magnitude of the induced immune activation and therefore the outcome of super antigen-mediated diseases [72].

The fucosyltranferase 2 (*FUT2*) gene is responsible for synthesizing ABH blood group antigens in the mucus and other secretions. Homozygous individuals for *FUT2* gene loss-of-function mutation show a non-secretor phenotype, which has been associated with an increased susceptibility of developing Crohn’s disease [73]; this mutation is also associated with CD [74]. In addition, FUT2 non-secretor status has been associated with increased serum lipase activity in asymptomatic subjects and an increased risk for chronic pancreatitis [75], a disorder strongly linked to CD [76]. A recent study described how *FUT2* genotype and *FUT2* gene expression could explain differences in gut microbiota composition. The non-secretor individuals were demonstrated to have an altered mucosa-associated microbiota in their intestinal tract, characterized by reduced diversity, richness and abundance of *Bifidobacterium spp.*, a bacterial genus that may play an important role in autoimmune disease risk [77,78,79]. To better understand CD etiology, the CD genetic component has been extensively studied by performing GWAS and the Immunochip study [7]. Currently, it is well-established that 39 non-MHC loci are also associated with the risk of developing CD. Some of these 39 non-MHC loci harbor genes related to bacterial colonization and sensing and would, therefore, be potential candidate loci to investigate the possible interactions between the gut microbiota composition and host genotype. Furthermore, other candidate loci are those harboring disease-associated single nucleotide polymorphism (SNPs) with the potential to develop regulatory roles in the expression of genes related to microbiota handling.

## 5. Epigenetics and Intestinal Microbiota: A New Emerging Field

Nowadays, it is well established that there are changes in gene expression or cellular phenotype triggered by epigenetic modifications, such as methylation or non-coding RNAs (ncRNAs), defined as RNA molecules transcribed from DNA but not translated into proteins. These are involved in post-transcriptional regulation of gene expression, among others, and not caused by changes in the DNA sequence. Interestingly, blood DNA methylation patterns are associated with gut microbiota profiles [80] and a recent study has also indicated the relationship between microbiota and methylation level of the free fatty acid receptor 3 gene, involved in metabolism and the inflammatory response [81]. Furthermore, methylation level at the IFNG locus is correlated with the immune response to microbial components and with the expression of IFN-γ in ulcerative colitis patients [82]. The relationship between ncRNAs and gut microbiota is a new research field. Until now, different studies have reported a link between miRNAs, a group of small ncRNAs, and microbiota [83]. Dalmasso *et al.* studied whether miRNAs are involved in microbiota-mediated regulation of host gene expression based on comparisons between germ-free mice and germ-free mice colonized with the microbiota from pathogen-free mice. They showed nine miRNAs differentially expressed in the ileum and colon of colonized mice compared to germ-free mice [84]. A similar study was performed by Singh *et al.* showing that the murine miRNA signature in the caecum is affected by the microbiota [85]. Moreover, authors found that 34 putative miRNA target genes encode for proteins involved in the regulation of the intestinal barrier function and immune response, indicating the interplay between microbiota and caecal miRNA signature [85]. Modifications of histone acetylation, related to local relaxation of the chromatin and access for transcription machinery by histone deacetylase (HDACs) are also critical in epigenomic regulation. HDACs are inhibited by commensal bacterial-derived SCFAs in innate and adaptive immune cell populations, suggesting that the metabolic activity of commensal bacterial can modify the epigenome of host cells and in turn alter their development and function [86]. In fact, SCFAs derived from commensal bacteria exert anti-inflammatory effects in the colon, partially by stimulating histone acetylation of Forkhead box P3 (FoxP3) locus in naïve CD4^+^ T cells and, thereby increasing FoxP3 expression and promoting the differentiation of Tregs [87]. However, research into the role of epigenetics in regulating the cross talk between the host and the microbiota is in the early stages, while studies related to CD have yet to be undertaken.

## 6. Environmental Factors and Intestinal Microbiota

Besides host genetics, environmental factors also influence microbiota composition; indeed, diet is one of the main drivers of gut microbiota composition and function [45,88,89,90,91]. The milk-feeding type (breast-milk *versus* formula) exerts an important effect on gut microbiota composition [91]. Breast milk promotes gut colonization by *Bifidobacterium* spp., leading to the association of this bacterial genus with the beneficial properties of infants’ health attributed to breast-feeding. Retrospective studies have shown that longer breast-feeding, and particularly, maintenance of breast-feeding when gluten is introduced, reduces the risk of developing CD or delays its onset [92]. However, subsequent prospective studies have not confirmed this protective effect of longer breast-feeding on CD [93,94]. These discrepancies could be related to the influence of additional confounding factors, which remain uninvestigated systematically as yet. In fact, a recent study has found that mothers with CD present a decrease in several immune markers IL-12p70, transforming growth factor (TGF)-β1 and secretory IgA (sIgA) and in numbers of *Bifidobacterium* spp. in breast-milk compared to healthy mothers [95]. Therefore, these differences in breast milk composition could be one of the additional factors influencing the protective effects of breast-feeding on infant health. Furthermore, wheat gliadins and other gluten peptides have been identified in breast milk using specific IgA-antibodies against gliadin, and the presence of gluten in breast milk may play a role in the induction of oral tolerance in breastfed infants [96]. Thus, it is tempting to speculate that the breast milk of mothers with CD following a GFD lacks this stimulus and other protective factors, which might influence the future gluten tolerance of their offspring. However, there are no robust prospective studies revealing how differences in breast milk composition and intestinal microbiota acquisition and evolution early in life might ultimately protect or contribute to CD onset.

The mode of delivery (vaginally or cesarean section) also has a strong influence on shaping the initial gut microbiota composition [97]. This is one of the perinatal and early postnatal environmental factors that clearly influences gut microbiota composition and is also associated with CD susceptibility [98]. The greater risk of developing CD in children born by elective caesarean section might be attributed to the delay in intestinal colonization by bifidobacteria, and the reduced bacterial diversity observed in caesarean-born compared to vaginally delivered infants [97]. 

GFD also seems to cause changes in the intestinal microbiota composition as well as in the immune response induced by the altered microbiota of immunocompetent cells *in vitro* [99]. In healthy adults, the GFD associated with a reduced intake of complex polysaccharides caused shifts in gut microbiota composition. Particularly, there were decreases in *Bifidobacterium* spp*.*, *Clostridium lituseburense* group, *Fecalibacterium prausnitzii*, *Lactobacillus* spp. and *Bifidobacterium longum* after adherence to a GFD, whereas *Escherichia coli*, *Enterobacteriaceae* and *Bifidobacterium angulatum* numbers increased [99]. Therefore, alterations detected in CD patients under a GFD could partly be due to the dietary effect and not only to the underlying disease.

Antibiotics and other commonly used drugs are also well known environmental factors exerting a profound impact on the microbiota composition, potentially modifying its functional role in health and disease [100]. Recently, a positive association between antibiotic exposure and CD development has been reported, as it has been the case for other inflammatory disorders [18]. This association suggests that perturbation of the microbiota by antibiotics may play a role in CD onset and pathogenesis.

## 7. Intestinal Dysbiosis and Its Potential Pathogenic Role in CD

Most observational studies in children and adults with CD have shown alterations in the intestinal microbiota composition compared to control subjects [21,22,101,102]. In this context, we performed studies using different quantitative methods to assess microbiota composition, such as fluorescence *in situ* hybridization (FISH) and quantitative PCR. Our results found reduced numbers of *Bifidobacterium* spp. and *B. longum* and increased numbers of *Bacteroides* spp. in stools and duodenal biopsies of CD patients, untreated and treated with a GFD, compared to control subjects [21,22]. We also found higher enterobacteria and staphylococci numbers in untreated CD patients compared with controls, but the balance was almost restored in CD subjects on a long-term GFD [21]. Likewise, other studies in children have reported an increased prevalence of *Bacteroides vulgatus* and *E. coli* in CD biopsies before and after GFD compared to controls, as well as lower numbers of *Lactobacillus* and *Bifidobacterium* and higher numbers of *Bacteroides, Staphylococcus* and enterobacteria in stools of children with CD compared to healthy controls [101]. Although there are ecological differences in the upper and lower part of the intestinal tract that influence the microbiota composition, our studies also showed that the alterations associated with CD were similar in both duodenal biopsies and fecal samples [21]. A study carried out by Schippa *et al.* analyzed the dominant mucosa-associated microbiota of duodenal biopsies by using temperature gradient gel electrophoresis (TTGE), revealing that the CD patients, before and after GFD, have a particular microbiota profile [103]. The authors also reported an increase in *Bacteroides vulgatus* and *Escherichia coli* in CD patients compared to controls [103]. Another analysis of proximal small intestine biopsies from 45 children with CD and 18 controls revealed that the microbiota from CD patients collected during the Swedish CD epidemic (2004–2007) differed only slightly from the microbiota found in controls currently. However, rod-shaped bacteria were found to constitute a significant fraction of the proximal small intestine microbiota in children born during the Swedish CD epidemic (1985–1996) detected by scanning electron microscopy and further analyzed by 16S rRNA gene sequencing, suggesting that such alterations could contribute to the fourfold increase in disease incidence at that time; nevertheless, the lack of similar associations in samples taken more recently (2004–2007) contradict this theory [104]. Other studies have analyzed the metabolites derived from intestinal microbiota activity, revealing significant differences between treated CD patients and healthy controls, suggesting there is a metabolic signature for the CD microbiome [102]. A very recent study has also reported that CD patients with gastrointestinal symptoms have different microbiota composition when compared with controls and patients with dermatitis herpetiformis, suggesting that the microbiota may play a role in the manifestation of the disease [24]. Furthermore, a dysbiotic microbiota seems to be associated with persistent gastrointestinal symptoms in treated CD, clearly indicating its pathogenic implication in these particular cases [105]. Nevertheless, we should also mention that other authors report no differences in mucosa-associated duodenal microbiome composition and diversity using a 16S–23S rRNA interspacer region-based profiling method [106] and there is lack of consensus and understanding of what constitutes a CD-promoting microbiota.

From the studies described above, it is still unclear whether the changes in the microbiota are a cause or a secondary consequence of CD development. The fact that intestinal dysbiosis has been observed not only in newly diagnosed CD patients but also in those treated with a GFD supports a primary role of gut microbiota in CD. Thus, it would seem that the microbiota are predisposed to CD, although the role of GFD in the microbiota alterations detected in treated CD patients cannot be disregarded [99].

A deeper characterization has been undertaken of the CD microbiota by isolating bacterial strains and analyzing their pathogenic features. Interestingly, *E. coli* clones belonging to virulent phylogenetic groups (B2 and D) isolated from untreated and treated CD patients present a higher number of virulence genes, encoding P fimbriae, capsule K5 and hemolysin, than those isolated from healthy controls [107]. A similar finding was reported by Schippa *et al.* in Crohn’s disease [43]. The authors characterized adhesive and invasive capabilities of *E. coli* strains found in adult and pediatric Crohn’s disease patients as well as in controls, and reported significant differences related to the disease. They identified particular *E. coli* variants (adherent invasive *Escherichia coli* strains) in the intestine of Crohn’s disease patients, suggesting that these could be generated via evolutionary phenomena driven by a persistent inflammatory state [43]. Furthermore, the abundance of *Bacteroides fragilis* strains coding for metalloproteases is increased in both untreated and treated CD patients, and this strongly supports a pathogenic role of intestinal dysbiosis and specific pathobionts in CD [108]. In fact, *Bacteroides fragilis* and, particularly, the strains producing metalloproteases are frequently involved in opportunistic infections and they aggravate colitis in animal models [109]. The isolation and identification of clones belonging to the genus *Staphylococcus* also revealed that *S. epidermidis* carrying the *mecA* gene (methicillin resistant gene) was more abundant in the CD patients (treated and untreated) than in controls [110].

**Figure 2:**
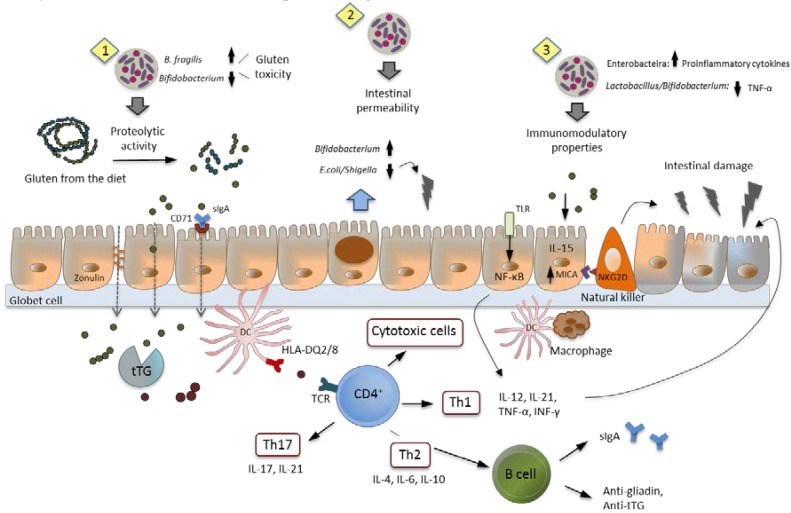
Potential mechanisms of action of intestinal microbiota components in CD. Schematic representation of CD pathogenesis and the potential role of intestinal dysbiosis. Some gluten peptides cross the intestinal epithelium and can be deamidated by the tissue transglutaminase (tTG), which increases their ability to bind the HLA-DQ2/8 molecules of antigen-presenting cells and to trigger an adaptive immune response, involving Th1, Th2 and Th17 cells. This leads to the release of pro-inflammatory cytokines (IFN-γ, interleukin (IL)-21, *etc.*) and the production of CD antibodies. Other gluten peptides activate the innate immune response by interacting with epithelial cells and APCs and, thus, triggering the activation of inflammatory pathways (NFκB) and the production of inflammatory cytokines such as IL-15. In particular, IL-15 increases the expression of the MICA molecule at epithelial cell surface and triggers activation of intraepithelial lymphocytes through engagement of NKG2D, leading to an innate-like cytotoxicity toward epithelial cells and enhanced CD8 T cell-mediated adaptive response, contributing to villous atrophy. The microbiota could contribute to the etiopathogenesis of CD by (2) providing proteolytic activities that influence the generation of toxic and immunogenic peptides from gluten and by mediating host-microbe interactions which could influence (1) the intestinal barrier and (3) immune function (e.g., via regulation of the cytokine network of pro-inflammatory and anti-inflammatory factors). Adapted from [114].

Different study models have also indicated the possible mechanisms of action of intestinal dysbiosis in CD (Figure 2). Specific alterations in the microbiota could contribute to the etiopathogenesis of CD by providing proteolytic activities that influence the generation of toxic and immunogenic peptides from gluten, and compromise the intestinal barrier function. In general, some gluten peptides (gliadin) partially resist gastrointestinal digestion and disrupt the intestinal integrity by altering the expression or localization of tight junction proteins and increasing epithelial permeability. In this respect, the microbiota may facilitate the access of gliadin peptides to the lamina propria and its interaction with infiltrated lymphocytes and APCs responsible for triggering the immune response via different mechanisms. *In vitro* studies indicate that the proteolytic activity of the intestinal microbiota may modify gliadin peptides differently, increasing or reducing their toxicity. *Bacteriodes fragilis* clones isolated from the intestinal microbiota of CD patients showed gliadin-hydrolyzing activity, and some of them generated peptides that maintain their immunogenicity, eliciting inflammatory cytokine production by Caco-2 cell cultures, and showing a greater ability to permeate the Caco-2 cell monolayer [108]. In contrast, different bifidobacteria and, particularly, *B. longum* CECT 7347 (also termed *B. longum* IATA-ES1) reduced the cytotoxic and inflammatory effects of gliadin peptides generated during gastrointestinal digestion [111]. Regarding the mechanism of action on the intestinal barrier function, CD-triggers (gliadin and IFN-γ) decreased the goblet cell numbers in intestinal loops of inbred Wistar-AVN rats, and enterobacteria isolated from CD patients, such as *Escherichia coli* CBL2 and *Shigella* CBD8, aggravated this effect [112]. Furthermore, exposure to these enterobacteria caused increased mucin secretion and greater disruption of tight junctions. By contrast, *Bifidobacterium bifidum* CECT 7365 (also named *B. bifidum* IATA-ES2) increased the number of goblet cells and the production of metalloproteinase inhibitors, and reduced gliadin translocation to the lamina propria, which could contribute to gut mucosal protection [112]. Other probiotic bacteria such as *Lactobacillus rhamnosus* GG contributed *in vitro* to the maintenance of normal intestinal permeability in Caco-2 cell cultures exposed to gliadin [113].

The composition of the gut microbiota also seems to influence the release of pro-inflammatory cytokines triggered by gluten peptides. For instance, a mixture of bacteria isolated from CD patients during the Swedish CD epidemic (*Prevotella* spp., *Lachnoanaerobaculum umeaense* and *Actinomyces graevenitzii*) induced IL-17A mRNA expression in *ex vivo* biopsies of intestinal mucosa of CD patients [115]. Thus, researchers have hypothesized that those bacteria could contribute to breakdown in gluten tolerance by increasing the IL-17 response. By contrast, in gliadin-sensitized HLA-DQ8 transgenic mice, a strain of *Lactobacillus casei* reduced the TNF-α levels in jejunal tissue sections [116]. In a model of newborn rats sensitized with IFN-γ and orally administered gliadin, *B. longum* CECT 7347 reduced TNF-α and increased IL-10 in intestinal tissue samples [117].

On the one hand, *B. longum* CECT 7347 and *B. bifidum* CECT 7365 reduced the inflammatory cytokine secretion (IFN-γ and TNF-α) induced by the fecal microbiota of CD patients while, on the other, they increased IL-10 secretion in peripheral blood mononuclear cell cultures [118]. *Escherichia coli* CBL2 and *Shigella* CBD8 isolated from CD patients, boosted the production of IL-12 and IFN-γ, and the expression of HLA-DR and CD40 in co-cultures of monocyte-derived dendritic cells (MDDCs) and Caco-2 cells compared to *B. longum* CECT 7347 or *B. bifidum* CECT 7365 [119].

## 8. Role of Probiotics in CD: Human Intervention Studies

The potential use of probiotics in CD management is supported by the intestinal dysbiosis generally associated with CD and the role attributed to these potentially beneficial bacteria (*i.e.*, “probiotics”) in maintaining gut barrier function and regulating the response of the innate and adaptive immune system. Based on this hypothesis, three randomized, double-blind placebo-controlled human intervention trials have been conducted in CD patients to date [120,121,122]. In one of these interventions, *B. infantis* NLS was administered to untreated CD patients to evaluate the effect of the probiotic independently of the GFD. This study reported an improvement in some gastrointestinal symptoms, specifically indigestion and constipation, in untreated CD patients after the administration of *B. infantis* NLS. Furthermore, it did not improve diarrhea or abdominal pain nor modify intestinal permeability or the pro-inflammatory status measured as the serum level in some cytokines and chemokines [120]. Another intervention study evaluated the influence of *B. longum* CECT 7347 in CD children on a GFD in order to assess whether this bifidobacteria probiotic could improve the efficacy of the GFD. This trial revealed a decrease in peripheral CD3+ T lymphocytes and a trend in the reduction of TNF-α serum levels after *B. longum* CECT 7347 administration, and also a relevant reduction of *Bacteroides fragilis* numbers and sIgA in stools when compared to the placebo group [121]. A recent three-month trial has also evaluated the effect of combining the strains *B. breve* BR03 and *B. breve* B632, as compared to a placebo, in children with CD on a GFD. The study reported that *B. breve* strains decreased the production of the pro-inflammatory cytokine TNF-α in children with CD on a GFD [122].

## 9. Concluding Remarks and Future Perspectives

To date, different studies have demonstrated associations between intestinal dysbiosis, CD and gastrointestinal manifestations of the disease. Microbiota imbalances have been observed not only in untreated CD patients but also in patients following a GFD. In addition, specific bacterial strains isolated from patients with active and non-active CD have been shown to have increased virulence features. These findings suggest that microbiota alterations are not only a mere consequence of the inflammatory status characteristics of the active phase of the disease. These alterations could play both a secondary role by aggravating CD pathogenesis and generating a vicious-circle, and a primary role by contributing to disease onset. Prospective studies in healthy infants at family risk of CD are also underway to decipher the co-evolution of the gut microbiome and the host genome in response to environmental factors and possible causal relationships with CD onset. We expect that CD results from the combination of an altered human genome and microbiome in conjunction with as yet unknown epigenetic modifications, partly due to different environmental factors, which together influence mucosal gene expression and the mucus layer, prompting self- and gluten reactivity in the host. Future progress in this area will be crucial to provide new clues to help improve CD management and primary prevention. This will also help us progress beyond the obscure scenario of unsuccessful intervention trials focusing only on the inclusion of gluten in the infant’s diet.
